# Genetic Predisposition and Genetic Resilience Factors in Stress-Related Disorders During the Developmental Age: A Narrative Review

**DOI:** 10.3390/genes16111362

**Published:** 2025-11-10

**Authors:** Alessia Raffagnato, Arianna Raicich, Lisa Paiusco, Giulia Coser, Ilaria Bonemazzi, Andrea Gazzin, Maria Federica Pelizza, Caterina Ancora, Irene Toldo

**Affiliations:** 1 Child and Adolescent Neuropsychiatric Unit, Department of Woman’s and Child’s Health, Padua Hospital, 35128 Padova, Italy; alessia.raffagnato@unipd.it (A.R.); arianna.raicich@aopd.veneto.it (A.R.); lisa.paiusco@aopd.veneto.it (L.P.); giulia.coser@aopd.veneto.it (G.C.); ilaria.bonemazzi@aopd.veneto.it (I.B.); mariafederica.pelizza@aopd.veneto.it (M.F.P.); 2 Department of Woman’s and Child’s Health, University of Padua, 35128 Padova, Italy; 3 Clinical Genetics Unit, Department of Integrated Diagnostic Services, University Hospital of Padova, 35128 Padova, Italy; andrea.gazzin@unipd.it; 4 Child Neuropsychiatry Unit, San Bortolo Hospital, ULSS 8 Berica, 36100 Vicenza, Italy; caterina.ancora@aulss8.veneto.it

**Keywords:** stress-related disorders, PTSD, acute stress disorders, adjustment disorders, reactive attachment disorders, genetic, epigenetics, resilience, children, adolescents

## Abstract

**Background:** Stress-related disorders, including PTSD, acute stress disorders, adjustment disorder, and attachment disorders, arise from complex interactions between genetic susceptibility and environmental stressors. While early environmental factors play a central role in the development of these disorders, there is growing evidence that genetic predisposition also contributes to individual differences in vulnerability and resilience. This narrative review examines current evidence on genetic predisposition and resilience mechanisms in stress-related psychopathology during developmental age. **Methods:** A literature search was performed using PubMed, Cochrane, MedRxiv, and Medline databases, focusing on studies published between 2010 and 2025, written in English, in the pediatric and adolescent population. Priority was given to original research articles and high-impact reviews. Studies were selected based on relevance to the genetic mechanisms underlying vulnerability and resilience to stress. 71 of 317 were selected. Two hundred forty-six articles were excluded due to a lack of relevance to the topic or because they included an adult population. **Results:** Polymorphisms and epigenetic modifications in genes involved in hypothalamus–pituitary–adrenal axis (*FKBP5*, *NR3C1*, *ADCYAP1R1* and *ACE*), serotoninergic (*SLC6A4* and *HTR2A*), noradrenergic and dopaminergic system (*COMT* and *MAOA*), *BDNF*, estrogen receptor and excitatory amino acid transporters are associated with increased risk of psychopathology following early trauma, but are also implicated in the development of resilience. **Conclusions:** Genetic factors influence both vulnerability and resilience to stress-related disorders. However, further studies based on the role of genetics are needed to advance precision and personalized medicine, which is still largely underexplored to this day in the field of stress-induced disorders.

## 1. Introduction

Stress-related disorders (SRDs) are heterogeneous conditions and can manifest with both mental and physical symptoms. The pathophysiology of SRDs involves multiple factors, including the nature of the stressor (observable or perceived) and an individual’s dynamically evolving vulnerability and maladaptation leading to imbalance. Additional variables, such as environmental context, culture, gender, and developmental stage, play a significant role [1].

According to DSM-5, Trauma- and Stress-Related Disorders include Reactive Attachment Disorder, Disinhibited Social Engagement Disorder, Post-Traumatic Stress Disorder (PTSD), Acute Stress Disorder, Adjustment Disorders, and Prolonged Grief Disorders [2,3,4]. These conditions are closely linked to other psychiatric disorders that may be associated with exposure to trauma, such as Borderline Personality Disorder [5], Major Depressive Disorder [3,6], suicidal behavior [6], and alcohol and substance use disorders [3,7]. It is well established that early life stressors and trauma significantly increase the risk of developing these mental disorders, not only in adulthood, but also during childhood and adolescence [6]. Existing evidence suggests that there are commonalities and differences in epigenetic and genetic profiling between adolescents and adults, with important individual variability [6].

According to several studies, the onset of mental SRDs depends on the interaction between genetic and environmental factors (GxE), especially the combination of biological vulnerabilities and the exposure to traumatic experiences during childhood [5,6,8]. GxE effects are statistical interactions that model the way in which genetic variants may influence the effect of environmental factors on a phenotype, including individual differences in response to trauma [8,9].

More recently, research has focused on the biological and neurobiological consequences of early life stressors to determine the molecular and cellular mechanisms underlying the vulnerability to mental disorders [3,7]. Emerging evidence suggests that psychosocial trauma can lead to epigenetic modifications, resulting in downstream effects on gene transcriptional regulation [3,9]. Notably, these epigenetic changes are passed down somatically from cell to cell and in some cases can be transmitted across generations, persisting even in the absence of the stressor [3,6,10,11]. In parallel, growing evidence suggests the role of genetic predisposition in shaping individual vulnerability to SRDs, especially during sensitive periods of neurodevelopment [7]. In this regard, key genes are involved in hypothalamus–pituitary–adrenal (HPA) axis regulation [12], monoaminergic signaling [13,14], and neuroplasticity [15] and contribute to these differences.

Genetic factors and epigenetic differences play a significant role in shaping resilient responses to trauma and stress [16]. Resilience—the ability to adapt successfully in the face of stress and adversity—emerges from the complex interplay of multiple factors, including genetics, epigenetics, developmental environment, psychosocial factors, neurochemicals, and functional neural circuitry. These systems play a critical role in the development and modulation of resilience [16]. While research has identified various contributors to resilience in response to child maltreatments—such as higher cognitive ability, self-regulation, supportive family and community relationships [17]—there is currently limited evidence on whether, or how, these factors directly influence genomic regulation or epigenetic process [2].

Neural circuits are central to both resilience and vulnerability to SRD, with altered connectivity between key brain regions associated with low-resilience phenotypes. Two core networks, reward and fear circuits, are critical for adaptive responses to stress and social challenges [16].

The aim of this review is to summarize current findings on genetic and epigenetic alterations associated with early life stress in adolescents, explore their potential role in the development of psychiatric SRDs, and investigate how these mechanisms may also underlie resilience.

## 2. Materials and Methods

This narrative review focuses on existing evidence about genetic predisposition and resilience mechanisms in stress-related psychopathology during developmental age. The methodology follows established guidelines for conducting narrative reviews, focusing on a comprehensive exploration and critical analysis of the literature without imposing systematic limitations.

### 2.1. Search Strategy

The relevant literature was obtained using the PubMed (USA), Cochrane (UK), MedRxiv (USA), and Medline (USA) databases, focusing on studies published between 2010 and 2025 that complied with the Preferred Reporting Items for Systematic Reviews and Meta-Analyses (PRISMA) guidelines.

The search was carried out until 30 September 2025. The search terms comprised combinations of “trauma-related disorder”, ”stress-related disorder”, “resilience”, “genetic”, “genes”, “genomic”, “epigenetic”, and “gene environment interaction”. Various forms of SRD were considered, including “post-traumatic stress disorder” (“PTSD”), “acute post-traumatic stress disorder”, “acute stress reaction”, “attachment disorder”, and “adjustment disorder”. Boolean operators (AND/OR) were applied to refine the search results.

Studies were included in the review if they involved pediatric and adolescent subjects (<18 years of age) experiencing traumatic/adverse experiences during childhood and were selected based on relevance to the genetic mechanisms underlying vulnerability and resilience to stress.

English-written full-text original research articles and high-impact reviews were included. Exclusion criteria included textbooks, editorials, letters to the editor, quantitative meta-analysis, and articles not connected to the topic of our review, concentrated on adult populations, and not providing sufficient detail on the genetics of risk or prevention factors.

### 2.2. Data Extraction and Synthesis

**Figure 1:**
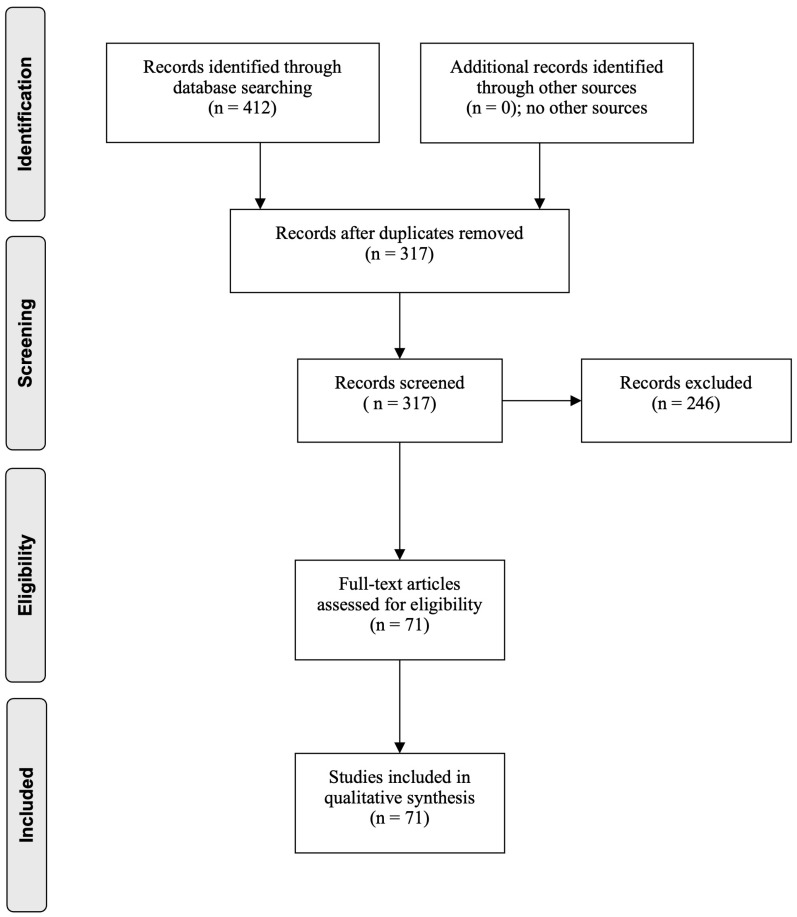
Flow diagram through the different phases of the review (PRISMA flowchart).

The results of the search process are summarized in Figure 1. Out of a total of 412 papers selected, 95 duplicates were removed. A total of 317 records were screened, and 71 full-text articles were assessed for eligibility and were included in the narrative review.

## 3. Genetic Predisposition and Epigenetics of SRDs During Developmental Age

The main results of the selected studies have been summarized in Table 1.

### 3.1. Genetic Predisposition to SRDs

An increasing number of studies highlight the influence of genetic predisposition on susceptibility to SRD during childhood and adolescence. Genetic factors contribute to individual differences in susceptibility to environmental stressors, particularly those occurring during critical periods of neurodevelopment. Key candidate genes identified thus far are involved in the regulation of the HPA axis, monoaminergic neurotransmission, and neuroplasticity, reflecting the multifaceted neurobiological mechanisms underpinning stress response and psychopathology.

#### 3.1.1. HPA Axis

The HPA axis is a neuroendocrine system that plays a pivotal role in regulating the body’s response to stress.

When a stressor is perceived, corticotropin-releasing hormone (CRH) is secreted by the hypothalamus, stimulating the pituitary gland to release adrenocorticotropic hormone (ACTH), which in turn triggers the adrenal cortex to produce cortisol and dehydroepiandrosterone (DHEA). Cortisol regulates the system through negative feedback on the hypothalamus and pituitary, while DHEA appears to counterbalance cortisol’s effects, exerting a protective, anti-glucocorticoid role [36,37].

The stress response system plays a crucial role in maintaining physiological balance (homeostasis) in the face of threat or change. However, when this system is chronically activated, it can have detrimental effects on the body, increasing the risk of developing psychiatric SRDs. Dysregulation of the HPA axis has been particularly implicated in various psychopathologies: elevated cortisol levels are frequently observed in major depressive disorder, whereas PTSD is often associated with reduced cortisol levels, which may act either as a predisposing factor or as a consequence of trauma exposure [7]. A growing body of research also indicates that early-life stress, such as childhood trauma, can lead to long-lasting alterations in HPA axis functioning, thereby affecting cortisol regulation and increasing vulnerability to stress-related phenotypes [38].

A key regulator of the HPA axis is *FKBP5*, a gene encoding a co-chaperone protein that modulates glucocorticoid receptor sensitivity and thus regulates the HPA axis negative feedback loop [18]. Several studies have consistently linked specific polymorphisms in *FKBP5*, such as rs1360780, to increased vulnerability to early life stress, including childhood maltreatment, neglect, and maternal trauma exposure [3,18]. *FKBP5* alleles interact with early life stress to increase the risk for PTSD [3,7,18].

The *NR3C1* gene, which encodes the glucocorticoid receptor, represents another central genetic target within the stress response system. Variants in *NR3C1* have been associated with altered HPA axis feedback sensitivity and deficits in emotion regulation [10]. Single-nucleotide polymorphisms (SNPs) in *NR3C1*—such as *rs41423247*, *rs10482605*, and *rs10052957*—are commonly included in genetic profile scores used to predict HPA axis function. These variants are suggested to be linked to an increased risk of volume alteration in regions such as the amygdala and hippocampus in children, as well as an increased risk for developing PTSD [10].

The Angiotensin I-converting enzyme (*ACE*) gene, which encodes a cell surface enzyme involved in the hydrolysis of circulating peptides, plays a key role in fluid and electrolyte balance, blood pressure regulation, and vascular remodeling. In addition to these physiological functions, *ACE* also contributes to the modulation of stress and fear responses by influencing HPA axis activity [26]. *ACE* D allele of *rs4311* may be linked to increased vulnerability to stress-related psychopathologies such as depression, PTSD, and anxiety disorders, potentially via enhanced HPA axis reactivity, but the results are controversial [26].

Several other genetic polymorphisms interacting with the HPA axis have been implicated in the modulation of stress responsivity and the emergence of psychopathology during development.

Polymorphism in *ADCYAP1R1* (adenylate cyclase activating polypeptide 1 receptor type I)—such as *rs2267735*—has been identified as a sex-specific genetic risk factor for PTSD, particularly in trauma-exposed children [4]. This gene is involved in the regulation of stress signaling pathways and neurodevelopmental processes [4]. A study on preadolescent children found that certain risk alleles of *ADCYAP1R1* were associated with increased PTSD symptoms in girls but not boys, suggesting a potential interaction between genetic risk, developmental stage, and sex hormones [4].

CRHR1 (corticotropin-releasing hormone receptor 1) plays a central role in initiating the stress response by regulating the release of ACTH, and its dysregulation has been linked to heightened risk for anxiety, depressive symptoms, and PTSD in youth, as well as differential sensitivity to early life stress [10,39]. This may influence the threshold for activation of the HPA axis in response to threat [10,39].

#### 3.1.2. Serotoninergic System

Genetic variations in several serotonin receptor genes have been shown to interact with environmental stress in shaping vulnerability to psychiatric disorders.

Serotonin turnover rises during acute stress in regions such as the amygdala, hypothalamus, and prefrontal cortex, with receptor subtypes exerting distinct effects [16,40]. For example, 5-HT1A receptors have anxiolytic properties, while 5-HT2A receptors appear anxiogenic, and 5-HT1B/2C receptors contribute to adaptive responses to stress [16,41,42].

An extensively examined gene is *SLC6A4*, which encodes the serotonin transporter (5-HTT). The *5-HTTLPR* polymorphism, in particular its short (s) allele genotype, has been linked to heightened emotional reactivity and increased susceptibility to develop bipolar disorder and depressive symptoms in youths exposed to trauma, including sexual and physical abuse, peer victimization, and familial conflict [27,28].

Further genetic variants related to serotonergic function, such as *HTR2A* (serotonin receptor 2A), have shown associations with stress sensitivity and depression, with evidence for both genetic and epigenetic contributions [13,19]. In particular, Vrshek-Schallhorn et al. conducted a longitudinal study harmonizing data from the Youth Emotion Project (387 young adults followed for 5 years) and a younger adolescent sample [13]. The study investigated how additive genetic risk within serotonin-system polymorphisms (*HTR1A*, *HTR2A*, *HTR2C*, *TPH2*) interacts with interpersonal stress to predict depressive diseases, supporting a polygenic additive model of psychopathology in the context of major interpersonal stress [13]. Interestingly, in the absence of such stress, carrying a higher genetic risk appeared to be a protective factor [13]. These findings suggest that serotonin-related variants may confer distinct environmental sensitivity, amplifying depression risk under stress but not necessarily in its absence. Such results were replicated in the adolescent sample, strengthening their relevance across developmental stages [13].

#### 3.1.3. Noradrenergic and Dopaminergic System

The noradrenergic system, centered in the locus coeruleus, orchestrates stress responses through projections to the amygdala, hippocampus, hypothalamus, and prefrontal cortex [36,43]. Hyperactivation of this system has been linked to the consolidation of negative emotional memories and to chronic anxiety, while pharmacological blockade of norepinephrine activity may attenuate such effects [44,45]. Imaging studies further suggest that disinhibited norepinephrine signaling enhances amygdala responsiveness to fear, contributing to PTSD pathology [46].

Polymorphisms in COMT (catechol-O-methyltransferase), a key enzyme involved in the degradation of dopamine in the prefrontal cortex, have been shown to influence cognitive control and emotional processing under stress [8]. The common *Val158Met* polymorphism of COMT is of particular interest, with the *Met* allele being associated with reduced enzymatic activity, increased dopaminergic tone, and greater stress sensitivity and risk for internalizing disorders in adolescence [8].

Monoamine Oxidase A (*MAOA*) encodes an enzyme involved in the catabolism of norepinephrine, serotonin, and dopamine. Early-life adversity, such as maternal separation or maltreatment, can induce long-lasting alterations in these neurotransmitter systems, potentially predisposing individuals to aggressive behavior and affective dysregulation [47].

Polymorphisms in the *MAOA* gene promoter region result in high (MAOA-H) or low (MAOA-L) enzymatic activity. Individuals with the MAOA-L variant may be at risk for blunted or dysregulated cortisol responses under stress, potentially reflecting impaired neuroendocrine adaptation [48].

Interestingly, sex differences have emerged in the behavioral and emotional effects of *MAOA* polymorphisms in adults. For instance, male carriers of MAOA-H exhibit increased dopamine release and heightened aggressive responses after exposure to violent stimuli, whereas female carriers of MAOA-H show higher vulnerability to burnout and depression following stressful life events [49,50].

#### 3.1.4. BDNF

Hippocampal Brain-Derived Neurotrophic Factor (BDNF) expression supports adaptive responses to chronic stress [16,51]. BDNF, expressed in regions such as the amygdala, hippocampus, prefrontal cortex, and basal forebrain, plays a crucial role in neuronal development, survival, and synaptic plasticity [52]. As shown by Sonoyama et al., loss-of-function mutation in the *BDNF* gene and its receptor TrkB impairs excitatory synaptogenesis in the hippocampus and is mechanistically linked to a broad spectrum of neurobehavioral abnormalities, including cognitive impairments, memory deficits, and phenotypes associated with neuropsychiatric disorders [53]. Moreover, the *BDNF* gene, essential for neuronal plasticity and development, has been extensively linked to psychiatric disorders in the adult population, especially PTSD and depression [54,55].

Some studies suggest an involvement of the BDNF, especially for the *Val66Met* polymorphism, in depression and PTSD among children and adolescents exposed to stressful life events; however, the findings remain inconsistent [14,30,31,32].

#### 3.1.5. Estrogen Receptor

Growing evidence points to estrogen receptor alpha 1 (*ESR1*), a gene involved in the nuclear regulation of estrogen signaling, as a sensitivity modulator to early life stress and stress-related consequences in youth. Variants in *ESR1* were found to attenuate the impact of stress in adolescents with depressive symptoms [13]. Mikhailova revealed a statistically significant difference in allele frequency for rs6557168 in *ESR1* by comparing different populations of adolescents born before, during, or after the 1991–1998 Russian socioeconomic crisis [26]. In her assessment, no such difference was identified in other commonly stress-related genes, including *MAOA*, *FKBP5*, *SLC6A3*, and *OXTR*, although it is important to note that the study did not correlate genetic variants to clinical phenotypes [26].

#### 3.1.6. Excitatory Amino Acid Transporters (EAAT)

The Solute Carrier Family 1 Member 3 (*SLC1A3*) encodes a glutamate transporter known as EAAT1, predominantly expressed in the central nervous system, playing a central role in the prevention of excitotoxicity through glutamate clearance and maintenance of synaptic neurotransmitter balance [56]. In the literature, *SLC1A3* mutations have been linked with neurodevelopmental disorders, especially Attention-Deficit/Hyperactivity Disorder (ADHD) [57].

A case–control association study was performed on more than 100 Indian adolescents (aged 16–19), presenting variable levels of clinically diagnosed stress and depressive symptoms, matched with more than 200 controls [14]. In this population, the T-allele of *SLC1A3 C3590T* was found to be associated with increased risk of both stress and depression [14], whereas other variants in *SLC1A3* and *BDNF* were not associated.

In parallel, genome-wide association studies (GWAS) have begun to uncover novel loci associated with PTSD and related conditions in trauma-exposed pediatric populations. However, the field faces challenges due to the heterogeneity of clinical phenotypes, limited sample sizes, and varying trauma assessments, resulting in partially inconsistent findings [58]. Nonetheless, these studies collectively support a model of complex, polygenic liability interacting with environmental stressors to shape mental health trajectories during development.

### 3.2. Epigenetics of SRDs

Epigenetic refers to heritable changes in gene expression that do not modify the underlying DNA sequence. They can be influenced by different environmental factors, including stress and early adverse experiences. Key mechanisms include DNA methylation, non-coding RNAs, and histone modification, which can exert long-term and even transgenerational effects influencing stress response and psychiatric vulnerability [59,60]. Epigenetic processes recently emerged as key mechanisms in the etiology of neurodevelopmental disorders, and various genetic conditions affecting neurodevelopment have been associated with specific methylation patterns [61,62].

Specifically, methylation implies the addition of methyl groups to DNA, usually at CpG sites, leading to a reduction in gene expression; histone modifications include changes to histone proteins influencing gene accessibility; non-coding RNAs such as microRNAs (miRNA) act as gene expression regulators after transcription [63].

Growing evidence links stress exposure (including trauma, physical, emotional, sexual abuse, neglect, bullying, violence), particularly in early life, with epigenetic alterations that can alter neurodevelopment. Epigenetic changes during these critical periods act as a bridge between genetic predisposition and environmental factors, contributing to the emergence of SRD, as well as other psychiatric conditions. In addition, the literature suggests a long-term negative impact on global health, influencing the risk for chronic organic disorders and cancer [11,64]. Childhood maltreatment has been frequently associated with epigenetic alterations in genes regulating the HPA axis. For example, methylation changes of the *glucocorticoid receptor* gene (*NR3C1*) observed in adulthood have been consistently associated with exposure to early-life adversity and are increasingly recognized as a potential epigenetic mechanism mediating the development of psychiatric SRD and suicidal behavior [65,66,67].

Longitudinal and population studies reinforce these findings. The TRAILS study, investigating the effects of stressors on 468 adolescents, found higher *NR3C1* methylation levels in those who had been exposed to life stress and adverse events during childhood and adolescence, notably without a significant correlation with perinatal stress [21]. They also discovered higher methylation of *SLC6A4* after stressful life events in adolescence, with a more pronounced association than during childhood [29].

Similarly, Romens et al. observed that children exposed to physical maltreatment displayed higher methylation levels in the *NR3C1* promoter compared to controls [22]. Notably, hypermethylation affected a precise part of the gene within the NGFI-A binding region, a critical site for both brain development and stress regulation [22].

Differential *NR3C1* methylation patterns were directly linked to psychopathology in a longitudinal study involving 487 children (mean age 12) by Bosmans et al. [23]. They demonstrated that higher *NR3C1* methylation increased vulnerability to developing anxious attachment when exposed to high stress and low maternal support, highlighting an epigenetic sensitivity to early caregiving environments [23].

In addition, a study by Efstathopoulos et al. investigated the relationship between epigenetic changes in *NR3C1* and internalizing symptoms in a population of Swedish adolescents aged 13–14 years [24]. In females, *NR3C1* hypermethylation was cross-sectionally associated with elevated scores for internalizing symptoms evaluated through the Center for Epidemiologic Studies Depression Scale for Children [24]. Furthermore, those who reported experiences of being bullied or lacking friends showed higher *NR3C1* methylation levels [24].

Epigenetic modifications have also been documented in other key stress-regulatory genes.

Hecker et al. [35] identified distinct methylation patterns of the proopiomelanocortin gene (*POMC*), encoding a precursor protein involved in the regulation of the HPA axis, in Tanzanian children exposed to different degrees of abuse.

FK506 binding protein 5 (*FKBP5*) encodes a protein involved in the regulation of the HPA axis by modulating the glucocorticoid receptor sensitivity. Growing evidence implicates it in the development of SRD through genetic and epigenetic mechanisms. A study by Parade et al. investigated the longitudinal methylation of *FKBP5* among preschoolers exposed to early adversities and stress [68]. They found, consistently over time, low methylation levels in maltreated children [68]. A similar result was uncovered by Non et al., who studied children exposed to early institutionalization, who were shown to have lower methylation levels at *FKBP5* and *SLC6A4*, with levels being related to the duration of institutionalization [20].

Gender differences in epigenetic profiles have been reported in maltreated populations.

For example, differential methylation was found to be associated with gender in the study by Cicchetti et al. [25], which analyzed epigenetic changes in over 500 low-income children, half of whom had documented histories of maltreatment. A significant interaction between maltreatment and gender was observed in the *ALDH2* gene: maltreated boys showed increased methylation compared to non-maltreated boys, whereas maltreated girls showed decreased methylation compared to non-maltreated girls [25]. In the *ANKK1* gene, an overall gender effect was also identified: girls had higher methylation levels than boys, regardless of maltreatment status [25].

Additional research has identified epigenetic modifications in serotonergic system genes. Parade et al. found that early stress and psychopathology (PTSD and depressive symptoms) were associated with site-specific methylation of the *HTR2A* gene (serotonin receptor 2A) in preschoolers [19]. Methylation varied by both genotype and stress exposure, with genotype moderating the stress–methylation relationship, suggesting early adversity may influence serotonergic function through epigenetic mechanisms.

Beyond candidate gene approaches, epigenetic changes have also been observed in genome-wide studies. Differential patterns of whole-genome DNA methylation were investigated in a study comparing a small number of institutionalized children aged 7–10 years with a group of children raised by their biological parents in Russia [69]. Institutionalized children showed increased genome methylation, particularly in genes involved in immune response, brain development, neural signaling, learning, and memory [69]. Weder et al. identified methylation in three genes (*ID3*, *GRIN1*, *TPPP*) as predictors of depression in a cohort of maltreated children [34].

Papale et al. identified over 500 differentially methylated loci across 122 genes, many previously linked to stress-related pathways in cohorts of prepubescent girls exposed to varying levels of childhood adversity [70]. Using RNA sequencing, the authors found more than 1400 differentially expressed genes, including *FHL3* and *NPC2*, further supporting the hypothesis that early-life stress leads to widespread and functionally relevant epigenetic changes [70].

Furthermore, Sheerin et al. conducted an epigenome-wide association study in treatment-seeking adolescents to examine the relationship between PTSD symptom severity and DNA methylation [9]. The study identified two CpG sites showing hypomethylation associated with higher PTSD symptoms, as well as one differentially methylated region overlapping the *MOBP* gene, involved in myelin-related neural processes [9]. One CpG also mapped to *MAML3*, a transcriptional regulator [9].

Esposito et al. analyzed DNA methylation in a cohort of adolescents who had gone through international adoption from conditions of poverty and disadvantage compared to adolescents raised by their biological parents in adequately resourced environments [71]. They found significant differences in DNA methylation associated with adversity occurring in early childhood, while adversity experienced later in childhood did not show similar epigenetic alterations, highlighting early childhood as a time-sensitive epoch for the epigenetic effects of stress [71].

Together, these findings support a model in which early stress severity triggers complex epigenetic remodeling contributing to the risk of expression of SRDs. Specifically, PTSD symptom severity in adolescents has been linked to epigenetic variations in genes involved in neural signaling and plasticity.

Neuroimaging studies have linked trauma-associated DNA methylation changes to structural brain differences in regions that govern stress responsivity, particularly within the hippocampus, amygdala, and medial prefrontal cortex [72].

To investigate this relationship, Ensink et al. employed a combination of methylome-wide association studies and structural neuroimaging measures in two independent cohorts of children and adolescents (aged 8–18 years) diagnosed with PTSD, compared with a control group [33]. Their work revealed significant methylation differences in children with PTSD regarding multiple genes, including those related to glucocorticoid functioning and Tenascin-XB (*TNXB*) [33]. *TNXB* encodes a glycoprotein associated with the extracellular matrix with roles in cell migration and tissue remodeling, interacting with proteins also implicated in hippocampal synaptic plasticity. Furthermore, the study found that methylation of the *OLFM3* gene, encoding a neuronal protein involved in the development of microglia, was related to the volume of the anterior hippocampus in both cohorts, being reduced in youth with PTSD [33].

In contrast to these findings, the large-scale Environmental Risk Longitudinal Study, which assessed 2232 twins born in England and Wales at different ages (5, 7, 10, 12, and 18 years), did not provide strong support for the hypothesis that victimization in youth leads to robust changes in DNA methylation [15]. Although the study investigated several forms of abuse (physical, sexual, and emotional) as well as neglect, exposure to violence, bullying, and crime, only sparse and inconsistent associations were observed, suggesting that the relationship between early trauma and DNA methylation may be more subtle or context-dependent than previously hypothesized [15].

## 4. Genetic Factors and Epigenetics of Resilience During Developmental Age

Resilience refers to the ability to adapt effectively to stress and adversity, maintaining psychological and physical stability [16]. Factors that shape baseline mental health can be distinct from those influencing recovery, stress adaptation, or post-traumatic growth [17]. Recent research has begun to uncover biological, psychological, and developmental factors, including genetics and neurochemistry, that contribute to resilience [16].

The main results of the selected studies have been summarized in Table 2.

### 4.1. Genetic and Epigenetic Factors Related to Resilience

#### 4.1.1. HPA Axis

As previously reported, the HPA axis is a central neuroendocrine system involved in coordinating the body’s response to stress.

It has been observed that children exposed to repeated stressful events display a blunted cortisol increase, suggesting a form of resilience [16,75]. Meanwhile, low DHEA or DHEA-sulfate (DHEA-S) levels have been connected to depression, and higher DHEA(S) levels have been noted in PTSD cases. Moreover, the DHEA(S)/cortisol ratio has emerged as a meaningful marker for stress vulnerability and resilience [16,80,81].

CRH and its receptors (CRHR-1 and CRHR-2) also play significant roles in stress regulation [44]. In contrast to CHRH-1, CRHR-2, found in regions such as the septum and dorsal raphe, may either amplify or reduce stress effects depending on the context [16,74,82].

Finally, as previously mentioned, ACE (angiotensin I-converting enzyme) also influences HPA activity. Conversely, the I allele, associated with lower ACE activity, has been proposed as a protective factor in some studies, potentially contributing to greater resilience, decreasing neuroendocrine and inflammatory stress responses. In adult population, the *ACE I*/*D* polymorphism may interact with environmental stressors in G × E models: individuals with the D/D genotype exposed to chronic stress may be particularly susceptible to HPA dysregulation and emotional dysregulation, while carriers of the I allele may maintain better HPA axis homeostasis under similar conditions, but the results are not univocal in a population of adolescents [26].

#### 4.1.2. Serotoninergic System

As previously discussed, multiple variations in serotonin transporters and receptors are involved not only in shaping vulnerability to psychiatric disorders but are increasingly recognized as contributing to mechanisms of resilience as well [16,41,42].

#### 4.1.3. Noradrenergic and Dopaminergic System

Both the norepinephrine transporter and adrenergic receptors (α and β) have been proposed as biological mediators of susceptibility and resilience to SRDs [16,83,84]. A key role is played by Catechol-O-Methyltransferase (COMT).

In accordance with the findings presented in Section 3.1.3 concerning the *Val158Met* polymorphism of the *COMT* gene, individuals homozygous for the Val allele (Val/Val) exhibit attenuated stress responses, suggesting a protective effect [8].

Another key gene in the stress response is monoamine oxidase A (*MAOA*). In contrast to what has been previously analyzed, female carriers of the 3-repeat (3R) low-activity allele have been associated with greater emotional stability and lower anxiety and depressive symptoms, suggesting sex-specific resilience profiles, even if results are not univocal [26].

#### 4.1.4. BDNF

Exogenous BDNF exerts antidepressant-like effects, enhances neurogenesis, and antidepressant treatments have been shown to upregulate BDNF and TrkB expression in the hippocampus and prefrontal cortex [16,85,86,87]. Still, some antidepressant effects appear independent of BDNF or neurogenesis [16,88,89]. The role of p75 signaling in resilience remains less clear, likely due to its low binding affinity [90]. The contribution of the *BDNF Val66Met* polymorphism to stress response and resilience remains uncertain.

La Greca et al. investigated how genetic vulnerability interacts with disaster-related stress exposure to predict PTSD and depression symptoms in children following a major natural disaster [76]. In this context, *BDNF* may represent one of several genes contributing to a broader neurobiological sensitivity to context, which either promotes resilience or enhances vulnerability depending on the quality and intensity of environmental input [76].

#### 4.1.5. NPY

Neuropeptide Y (NPY) plays a key role in stress regulation, exerting anxiety-reducing effects and supporting adaptive responses to adversity [40]. In animal models, reduced NPY expression was found in stress-related brain regions in PTSD-like conditions, while NPY supplementation reversed these effects [40,91]. NPY is widely expressed across key brain regions involved in emotional regulation, including the hypothalamus, amygdala, hippocampus, and locus coeruleus [16,40,92,93]. Genetic differences in the *NPY* gene have been linked to varying levels of stress resilience. For instance, certain SNPs have been associated with a higher risk of anxiety following early-life stress, likely due to changes in *NPY* expression and reduced HPA axis regulation [77]. Research has shown that genetic variants—particularly in the promoter region of the *NPY* gene—can influence NPY levels, with lower expression linked to reduced resilience [94,95]. It also counters the anxiety-inducing effects of CRH [93].

#### 4.1.6. Glutamate, GABA

Glutamate, GABA, and endocannabinoids have been strongly implicated in stress regulation, resilience, and the pathophysiology of mood and anxiety disorders [96,97,98]. Dysregulation within these systems can impair the ability to adapt effectively to both acute and chronic stress. Recent pharmacological studies targeting glutamatergic, GABAergic, and endocannabinoid signaling have yielded encouraging results, pointing to their potential as therapeutic avenues in psychiatric disorders [16,99,100,101]. As an example, *CNR1* (cannabinoid receptor type 1) codes for G protein–coupled receptor CB1R, ubiquitously expressed in the central nervous system and in peripheral neurons. Endocannabinoid signaling participates in neural plasticity, learning, and regulation of HPA response to acute or repeated stress. It is believed that the mechanism of development of stressor tolerance is based on the initiation of CB1R signaling caused by repeated stress, thus allowing the risk of negative consequences to be reduced, although results are controversial [26,102,103].

#### 4.1.7. OXTR

Several other genes are involved in influencing individual sensitivity to environmental contexts.

One of the most widely studied genes in this domain is the oxytocin receptor gene (*OXTR*), which plays a central role in social cognition and emotional regulation. A commonly examined polymorphism within *OXTR* is rs53576, characterized by an adenine (A) to guanine (G) substitution in the third intron [78]. This SNP has been associated with differential susceptibility to environmental influences, particularly in relation to family dynamics and early caregiving environments.

Individuals carrying the G allele have been found to exhibit higher levels of resilience and positive affect in environments characterized by high warmth and stability, but conversely, lower levels of resilience and well-being in contexts of low support or instability [16,73]. This pattern supports a differential susceptibility model, in which G allele carriers are more sensitive to the quality of their social environment. This heightened social sensitivity may also make G allele carriers more responsive to family-based preventive interventions, but also more vulnerable to the adverse effects of unsupportive parenting.

In line with this, Smearman et al. identified a significant three-way interaction between OXTR genotype, parenting quality, and intervention exposure in predicting telomere length, a biological marker of cellular aging and cumulative stress [78]. Specifically, GG individuals exposed to non-supportive parenting and randomized to the control group (i.e., no intervention) exhibited the shortest telomere lengths, suggesting increased biological stress [78]. In contrast, GG individuals who received the family-based intervention displayed telomere lengths comparable to low-risk groups, indicating a buffering effect of the intervention [78]. Notably, individuals with the A allele did not show significant differences in telomere length across conditions, suggesting reduced sensitivity to environmental variation.

The observed telomere shortening among GG individuals in adverse environments was partially mediated by chronic anger: those exposed to non-supportive parenting and not receiving the intervention showed greater increases in chronic anger over time, which in turn was associated with accelerated telomere erosion [78]. These findings highlight a biological embedding of social experience, with OXTR genotype moderating the extent to which environmental factors shape long-term physiological outcomes.

Also, Mikhailova et al. examined allele distributions of several resilience-related genes, including *OXTR rs53576*, among Russian adolescents born before, during, and after the socioeconomic crisis of the 1990s, but their findings were not statistically significant [26].

Epigenetic regulation of *OXTR*, as DNA methylation at the promoter region, has emerged as a mechanism by which early life experiences become biologically embedded, influencing individual trajectories of stress responsivity and resilience. In fact, it has been found that higher levels of OXTR promoter methylation were significantly associated with greater exposure to early adversity, particularly maternal depression and harsh parenting. Importantly, increased methylation was also linked to elevated depressive symptoms, suggesting that *OXTR* epigenetic silencing may impair the stress-buffering capacity of oxytocin signaling [78]. These findings support a biological pathway by which early social environments influence long-term emotional functioning through epigenetic downregulation of oxytocin signaling. Reduced *OXTR* expression may weaken the capacity for social affiliation and emotional regulation, both of which are essential components of psychological resilience. Moreover, the interaction between *OXTR* methylation and early stress may serve as a biomarker for susceptibility to affective disorders in adolescence and beyond. In contrast, lower methylation levels (potentially reflecting greater OXTR expression) have been associated with more adaptive emotional responses, increased social support seeking, and better outcomes following stress exposure, indicating a potential resilience phenotype. This aligns with broader evidence implicating the oxytocinergic system in the modulation of social buffering, especially in contexts of early life adversity [79].

Together, these findings highlight how epigenetic mechanisms mediate the impact of stress on the brain, influencing resilience and susceptibility to psychiatric disorders.

Nonetheless, it is worth noting that most studies on epigenetics and resilience factors are conducted on animals and adult populations.

## 5. The Role of Psychology and Environment in Coping with Stress

Several psychological and behavioral characteristics contribute to resilience in the face of adversity. High cognitive functioning, effective self-regulation, optimism, active coping, secure attachment, and social connectedness are consistently protective [104]. Positive emotions can also attenuate the expression of genetic vulnerability, such as BDNF Val66Met polymorphism or family history of depression [17,105]. Tendency to experience positive emotions shows moderate heritability (~0.60), but environmental factors, including parenting and daily experiences, strongly shape their expression [17,106,107,108].

## 6. Conclusions

In conclusion, studies investigating genetic and epigenetic modifications associated with early life stressors and the onset of psychiatric disorders in adolescents are still limited. The impact of genetic and epigenetic factors on the development of SRDs is of considerable relevance, particularly during adolescence, which represents a critical window in which heightened neural plasticity and ongoing epigenetic programming may amplify the effects of early adversity.

Evidence indicates that polymorphisms and epigenetic alterations in genes involved in the HPA axis (such as *FKBP5*, *NR3C1*, *ADCYAP1R1*, *ACE*), serotoninergic system (*SLC6A4*, *HTR2A*), noradrenergic and dopaminergic pathways (*COMT*, *MAOA*), as well as BDNF, estrogen receptors and excitatory amino acid transporters, are associated with an increased risk of psychopathology following early trauma, but are also implicated in mechanisms of resilience. It should also be noted that most of these variants are common polymorphisms, generally considered benign, and their individual contribution to psychiatric SRDs appears modest, making the concept of a strong gene × environment interaction still debated. Understanding the psychological substrates and neurobiology underlying factors implicated in coping with stress may help develop strategies aimed at preventing psychopathology after exposure to severe adversity. Furthermore, the mechanisms through which these epigenetic modifications contribute to psychiatric vulnerability—or conversely to resilience—during adolescence are not yet fully understood. Current evidence suggests both overlaps and differences in genetic and epigenetic profiles between adolescents and adults, with substantial interindividual variability. Nonetheless, genetic factors appear to shape both vulnerability and resilience to SRDs. A deeper understanding of gene–environment interactions, and of the neurobiological and psychological substrates involved in stress coping, holds promise for improving prevention strategies and therapeutic interventions. However, further research is needed to translate these findings into precision and personalized medicine, which remains largely underdeveloped in the field of stress-induced disorders.

## Figures and Tables

**Table 1:** Genetic and epigenetic predisposition to SRDs in Children and Adolescents.

Gene	Genetic or Epigenetic Changes	Stress-Related Psychiatric Disorder	Reference
***FKBP5*** (FK506-Binding Protein 5)	Polymorphism rs1360780	PTSD, Depression, Alcohol Use Disorders (AUD)	[3,7,18]
	Demethylation	PTSD	[7,19,20]
***NR3C1*** (Glucocorticoid Receptor)	Polymorphisms rs41423247, rs10482605, rs10052957	PTSD	[10]
	Hypermethylation	Anxiety, Emotional Lability, Externalizing and Internalizing Symptoms	[21,22,23,24,25]
***ACE-I*** (Angiotensin I-Converting Enzyme)	Polymorphism ACE D allele of rs4311	Depression, Anxiety, PTSD	[26]
***ADCYAP1R1*** (Type I Adenylate Cyclase Activating Polypeptide Receptor)	Polymorphism rs2267735	PTSD	[4]
***CRHR1*** (Corticotropin-Releasing Hormone Receptor 1)	Polymorphism rs4792887, rs110402, rs242941, rs242939, rs1876828	Depression, Anxiety, PTSD	[10]
***SLC6A4*** (Solute Carrier Family 6 Member 4)	Polymorphism 5-HTTLPR	Depression, Bipolar Disorder	[27,28]
	Methylation	Externalizing behavior	[20,29]
***HTR2A*** (Serotonin Receptor 2A)	Polymorphism HTR1A, HTR2A, HTR2C, TPH2	Depression	[13,19]
***COMT*** (Catechol-O-Methyltransferase)	Polymorphism Val158Met (rs4680)	Psychosis	[8]
***MAOA*** (Monoamine Oxidase A)	Polymorphism MAOA-L and MAOA-H	Aggressivity (male) Depression (female)	[26]
***BDNF*** (Brain-Derived Neurotrophic Factor)	Polymorphism Val66Met	Depression, PTSD	[14,30,31,32]
	Methylation	PTSD, Depression, Suicidal behavior	[11]
***ESR1*** (Estrogen Receptor Alpha)	Polymorphism rs6557168	Anxiety, Suicidal behavior	[26]
***SLC1A3*** (EEAT1—Excitatory Aminoacid Transporter 1)	Polymorphism C3590T	Depression, Stress, ADHD	[14]
***TNXB*** (Tenascin XB)	Hypermethylation	PTSD	[33]
***MOBP*** (Myelin Associated Oligodendrocyte Basic Protein)	Methylation	PTSD	[9]
***TPPP*** (Tubulin Polymerization Promoting Protein)	Methylation	Depression	[34]
***GRIN1*** (Glutamate Receptor, Ionotropic N-methyl-D-aspartate 1)	Methylation	Depression	[34]
***ID3*** 3 (DNA-Binding Protein Inhibitor ID–3)	Methylation	Depression	[34]
***POMC*** (Proopiomelanocortin)	Methylation	Emotional and behavioral problems	[35]

**Table 2:** Genetic and Epigenetic Factors of Resilience in Children and Adolescents.

Gene	Genetic or Epigenetic Changes	Stress-Protective Effects	Reference
***CRHR1*** (Corticotropin-Releasing Hormone Receptor 1)	Allelic variants	Controversial role in the regulation of stress reward	[16,73]
***CRHR2*** (Corticotropin-Releasing Hormone Receptor 2)	Polymorphism	May amplify or reduce stress effects	[16,74]
***ACE-I*** (Angiotensin I -Converting Enzyme)	Polymorphism ACE I allele of rs4311	Decreases neuroendocrine and inflammatory stress responses (results not univocal)	[26]
***COMT*** (Catechol-O -Methyltransferase)	Polymorphism Val158Met	Influences the capacity to experience reward	[8,16,17,75]
***MAOA*** (Monoamine Oxidase A)	Polymorphism MAOA-L	Greater emotional stability, lower anxiety and depressive symptoms (female; not univocal results)	[26]
***BDNF*** (Brain-Derived Neurotrophic Factor)	Polymorphism Val66Met	Uncertainty contributes	[16,17,76]
***NPY*** (Neuropeptide Y)	Polymorphism	Anxiety-reducing effects, supports adaptive responses to adversity	[16,77]
***FKBP5*** (FK506-binding protein 5)	Single Nucleotide Polimorfism	Controversial role in coping with stress	[16]
***CNR1*** (Cannabinoid receptor type 1)	Polymorphism	Neural plasticity, regulation of HPA response (controversial results)	[26]
***OXTR*** (Oxytocin receptor gene)	Methylation	Influences the stress response	[78,79]
	Polymorphism (rs53576, G allele)	More sensitive to the social environment, influencing the response	[26,78]

## Data Availability

No new data were created or analyzed in this study. Data sharing is not applicable to this article.
